# Population, behavioural and environmental drivers of malaria prevalence in the Democratic Republic of Congo

**DOI:** 10.1186/1475-2875-10-161

**Published:** 2011-06-09

**Authors:** Jane P Messina, Steve M Taylor, Steven R Meshnick, Andrew M Linke, Antoinette K Tshefu, Benjamin Atua, Kashamuka Mwandagalirwa, Michael Emch

**Affiliations:** 1 Department of Geography, University of North Carolina, Chapel Hill, NC, USA; 2 Carolina Population Center, University of North Carolina, Chapel Hill, NC, USA; 3 Department of Epidemiology, Gillings School of Global Public Health, University of North Carolina, Chapel Hill, NC, USA; 4 Division of Infectious Diseases and International Health, Duke University Medical Center, Durham, NC, USA; 5 Department of Geography, University of Colorado at Boulder, Boulder, CO, USA; 6 Ecole de Santé Publique, Faculté de Médecine, Université de Kinshasa, République Démocratique du Congo; 7 Programme National de Lutte contre le Paludisme (PNLP), Kinshasa, République Démocratique du Congo; 8 Kinshasa General Hospital (HGK), Kinshasa-Gombe, DRC

## Abstract

**Background:**

Malaria is highly endemic in the Democratic Republic of Congo (DRC), but the limits and intensity of transmission within the country are unknown. It is important to discern these patterns as well as the drivers which may underlie them in order for effective prevention measures to be carried out.

**Methods:**

By applying high-throughput PCR analyses on leftover dried blood spots from the 2007 Demographic and Health Survey (DHS) for the DRC, prevalence estimates were generated and ecological drivers of malaria were explored using spatial statistical analyses and multilevel modelling.

**Results:**

Of the 7,746 respondents, 2268 (29.3%) were parasitaemic; prevalence ranged from 0-82% within geographically-defined survey clusters. Regional variation in these rates was mapped using the inverse-distance weighting spatial interpolation technique. Males were more likely to be parasitaemic than older people or females (p < 0.0001), while wealthier people were at a lower risk (p < 0.001). Increased community use of bed nets (p = 0.001) and community wealth (p < 0.05) were protective against malaria at the community level but not at the individual level. Paradoxically, the number of battle events since 1994 surrounding one's community was negatively associated with malaria risk (p < 0.0001).

**Conclusions:**

This research demonstrates the feasibility of using population-based behavioural and molecular surveillance in conjunction with DHS data and geographic methods to study endemic infectious diseases. This study provides the most accurate population-based estimates to date of where illness from malaria occurs in the DRC and what factors contribute to the estimated spatial patterns. This study suggests that spatial information and analyses can enable the DRC government to focus its control efforts against malaria.

## Background

Malaria is the vector-borne disease causing the most deaths in the world today, and is one of three principal causes of mortality in the Democratic Republic of Congo (DRC) [1,2]. Accurate estimates of the epidemiology and burden of malaria are lacking, but the World Health Organization (WHO) estimates that in 2006, malaria caused 247 million clinical cases globally, killing nearly one million people, primarily children in sub-Saharan Africa [3,4]. The Roll Back Malaria program estimates even more cases for 2006 at 300-500 million [5]. Additionally, malaria morbidity contributes substantially to disease burden by chronically debilitating tens of millions with symptoms such as severe anaemia [6]. In some endemic countries like the DRC, malaria accounts for up to 40% of public health expenditures and 30 to 50% of hospital admissions [3]. The emergence of highly drug-resistant parasites [7-11] underscores the need for prevention, and suites of preventive interventions have produced marked declines in malaria infections and mortality in several sub-Saharan African settings [12-22]. In highly malarious countries like the DRC, efficient intervention and preventive efforts must be guided by understanding the geographic patterns of prevalence and the factors underlying these patterns. Even in highly malarious countries like the DRC, malaria prevalence varies across space. Thus prevalence maps are needed in order to focus interventions in regions where they are most needed

Infection with malaria parasites is dependent on mosquito and human factors. Environmental factors such as land cover, rainfall, altitude and temperature affect Anopheles breeding and have been used to predict malaria transmission risk. Areas with greater amounts of precipitation and higher temperatures are expected to have greater malaria prevalence, as these conditions favour breeding of many Anopheline species as well as parasite reproduction within the mosquitoes [23-33]. Agriculture and urbanization may affect malaria transmission as well; highly cultivated areas have increased suitable habitat for most of the primary vectors, which are non-forest and prefer sunlight, while urbanized areas tend to have reduced vector breeding habitat, although decreased sanitary conditions in urban areas may promote vector breeding in some instances [34-39]. Conflict and warfare have also altered the local ecology of many parts of the DRC, leaving agricultural fields untended and susceptible to collecting water in which mosquitoes may breed [40]; however, a major focus of humanitarian efforts in war-affected regions is in preventing and treating malaria [41]. Human behaviours such as the use of bed nets and access to anti-malarial drugs are also vital for reducing risk.

Though the DRC is one of the most highly malaria-endemic countries in Africa [42] the limits and intensity of transmission within the country are unknown. For many infectious diseases in developing countries, data quality is poor because it is extrapolated from convenience samplings or non-random sentinel populations. Estimates of disease burden depend upon reliable prevalence data. While several studies have delineated endemic malaria zones in Africa, none have used population-based molecular diagnostics to produce detailed estimates of the spatial patterns and drivers of malaria prevalence. To this end, surveillance systems for infectious diseases in developing countries like the DRC can guide public health interventions at the sub-national scale. Demographic and Health Surveys (DHSs) are well established sources of population-based data on demography, reproductive health and HIV, and thus can provide unbiased prevalence data to enable better disease burden calculations and more well-informed allocation of resources [43]. This study uses specimens collected from the 2007 DRC DHS to provide estimates of malaria prevalence across the country.

## Methods

### Demographic and health surveys

**Figure 1:**
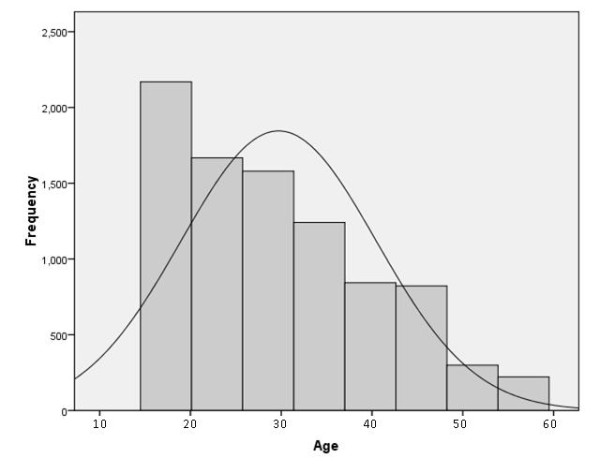
**Age distribution of the sampled population in the DRC DHS dataset displayed against the normal curve**. Mean age is 29.71 years (Standard Deviation = 10.755).

The DHS provides accurate demographic data in developing countries via large representative population-based surveys and in some countries also includes blood sampling for HIV surveillance. By leveraging the DHS infrastructure, molecular diagnostics for malaria were employed using leftover dried blood spots from the 2007 DRC DHS. Nine thousand households were surveyed. Of these, 9,000 households, 99.3% were successfully identified and interviewed. This included 4,757 men aged 15-59 years, all of whom were tested for HIV infection, as well as 9,995 women aged 15-59 years, half of whom were tested for HIV. The age distribution of the sampled respondents is presented in Figure 1. A chi-square test of age distribution by geographic cluster revealed no significant differences in the distribution between clusters (p = 0.343). Genomic DNA was extracted from the dried blood spots for testing in real-time PCR assays for *Plasmodium falciparum, Plasmodium malariae*, and *Plasmodium ovale *[44,45]. Malaria parasitaemia was thus determined for each individual who had been tested for HIV. The DHS was taken in urban Kinshasa during the rainy season (January 31-March 8, 2007). The remainder of the country was surveyed May-August, 2007 which is mostly dry season. Data on clinical symptoms are unavailable in the DHS database.

### Mapping of malaria prevalence in the DRC

Geographic coordinates of clusters of households were collected with global positioning system receivers. To ensure privacy, the coordinates of these 300 communities were randomly displaced by 5 km in rural areas and 2 km in urban areas. The number of respondents per community ranged from 14 to 53, with an average of 30. Malaria prevalence was computed for each community using the survey's sampling weights. A smoothed map of the spatial pattern of malaria prevalence in the DRC was then created in a geographic information system (GIS) using inverse distance weighting (IDW) spatial interpolation in ArcGIS 9.3 (ESRI, Redlands CA). IDW uses nearby values to predict prevalence in unmeasured locations. In our analysis, the prevalence values of the 12 closest communities to an unmeasured location were used to interpolate its prevalence value, with closer communities having a greater influence than those farther away. While other interpolation methods, such as kriging may be appropriate in certain instances, this method produces smoothed maps, eliminating areas of extremely high and low prevalence values from the interpolated surfaces. Inverse distance weighting maintains the entire probability distribution of prevalence values, for which we have authentic data to support.

### Assessing drivers of malaria prevalence in the DRC

**Figure 2:**
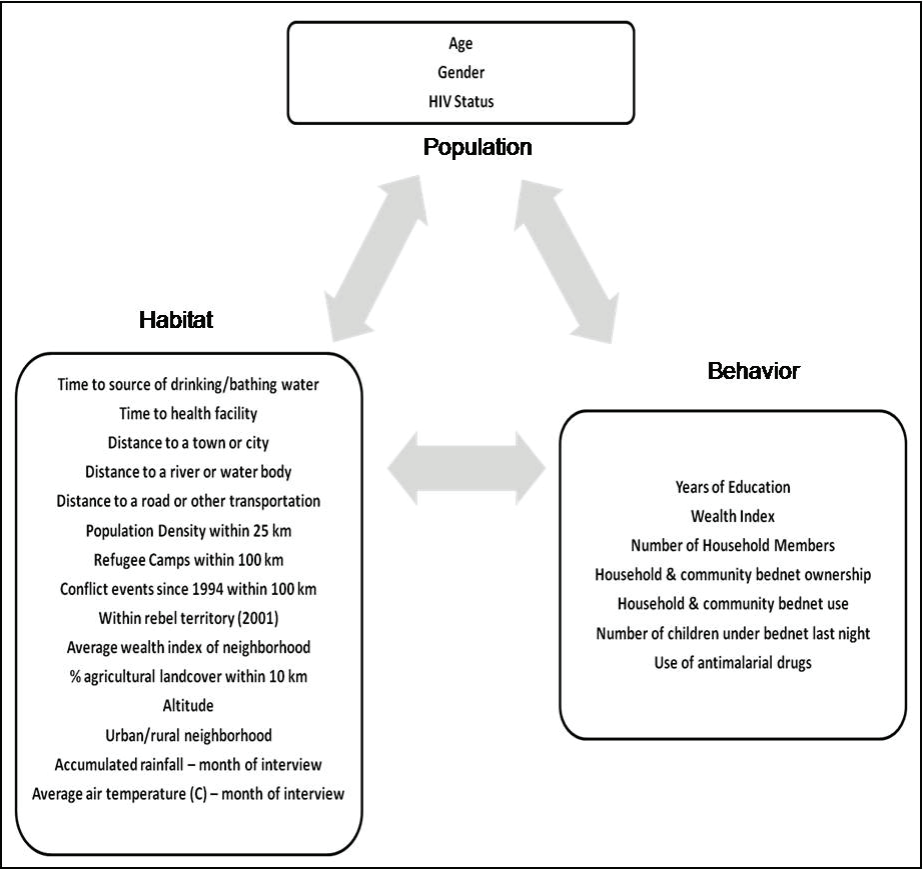
**Variables entered into multivariate analyses according to population, habitat, and behavioural characteristics**.

A multivariate analysis was conducted to estimate factors driving malaria prevalence in the DRC. A diagram of factors considered is provided in Figure 2 according to population, behaviour, and habitat/environmental categories.

### Ecological database creation

All population and behavioural variables were obtained from the DHS survey, as well as the time to a water source, time to a health facility, average wealth index and bed net use by community, altitude, and urban versus rural community type. The wealth index was computed by scoring households according to goods owned (television, radio, car, furniture, etc.) and lodging characteristics (electricity, drinking water, toilet type, roof material, cooking fuel type) using principal components analysis and then classifying the scores into quintiles, resulting in an index of 1-5 ranging from the poorest to the richest. The remaining habitat/environment variables were computed in the GIS. GIS layers for water bodies, roads, and cities were obtained from the Vector Map Level 0 (VMAP0) of the National Imagery and Mapping Agency (NIMA, 1997) and used to compute the distance from the community centroid to the nearest primary or secondary road, nearest river or major water body, and nearest city in kilometres. The same was done for towns (NIMA, 2003). Notably, while the VMAP0 dataset is the most recent available for determining the locations of roads, it is likely that in the face of economic collapse many of the roads have changed or disappeared since 1997.

**Figure 3:**
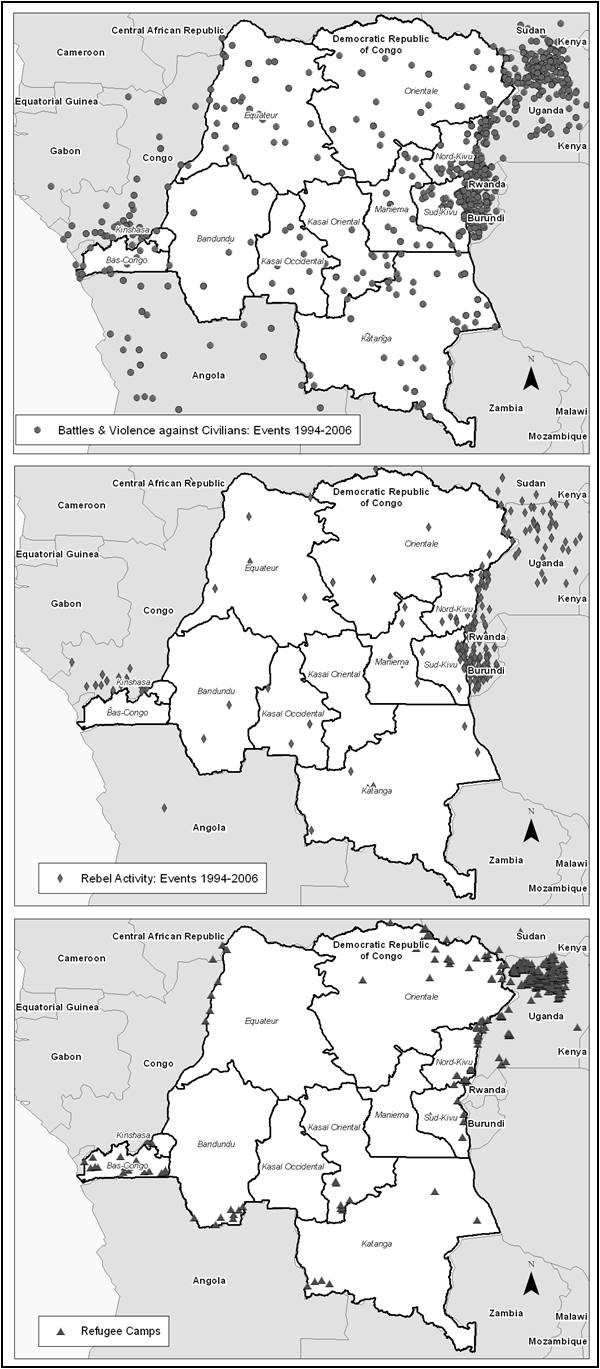
**Conflict and Refugee Camps in the DRC**.

A GIS database of armed conflict and refugee camp locations was also compiled in order to examine the effects of ongoing warfare in the DRC on malaria transmission. The Armed Conflict Locational Event Dataset (ACLED) includes locations and dates of individual battle events and rebel activity in states affected with civil war [46]. For the DRC and its surrounding countries, information dating from 1960 onward is available. Fighting in the eastern DRC increased in 1994, and conflict variables were computed between 1994 and 2006 (the year before the DHS survey was conducted). The variables used in this study included battle events within 100 km, rebel activities within 100 km, and all conflict events combined within 100 km. This distance was chosen as population migration from conflict is expected to occur across larger distances. The locations of current and recently closed (post-2004) refugee camps and settlements in the DRC and its surrounding countries were obtained from the United Nations Human Rights Council. Recently closed camps were included as they were likely still inhabited. The distance of a community centroid to a refugee camp was computed, as well as the density of camps within 100 km of the communities. Figure 3 shows the locations of battle events, rebel activity, and refugee camps as described here. A community's location within rebel territory as defined by Coghlan *et al *was also computed in the GIS [47,48]. Rebel territory was defined by these authors as of 2001.

Additional geographic variables included the accumulated rainfall (mm) at the community centroid for the month prior to interview and average air temperature (degrees Celsius) at the month of interview, both computed using Tropical Rainfall Measuring Mission data (TRMM) (NASA, 2009). The percent of agricultural landcover within 10 km of one's community (representative of the average Anopheles flight distance range) was computed using LANDSAT TM images from the years 2000-2001 which were classified using FAO/UNEP international standards by FAO Africover [49]. The population per square kilometer within 25 km of each survey community was also computed using a population density grid [50].

### Statistical methods

The population, habitat, and behavioural indicators diagrammed in Figure 2 were entered into a multilevel logistic regression model. Individual response variables related to age, gender, HIV status and behaviours were entered into the model along with the array of community-level variables. The dichotomous outcome variable was the presence or absence of any species of malaria parasite from real-time PCR testing. Multilevel analysis was chosen because the nested structure of the data required simultaneous examination of group- and individual-level variables [51,52]. Additionally, the multilevel approach produces correct standard errors and parameter estimates if outcomes for individuals within groups are correlated (and thus the standard regression assumption of independence of observations is violated). Conceptually, a multilevel model is a two-step set of equations, one explaining variation at the individual level, and the other explaining variation at the group level. Bivariate correlations between all variables were tested prior to entering variables into the model in order to avoid multicollinearity. The model was built in SAS v. 9.2 (SAS Institute, Cary, N.C.) using PROC GENMOD. The best-fitting model was chosen using Akaike's Information Criterion (AIC) which favours parsimony by making a trade-off between the precision and complexity of each model (lower AIC values are favoured).

The relationship between conflict and malaria prevalence was then tested with a geographically-weighted regression (GWR), which estimates local models for each community and its neighbours as defined by a spatial weighting matrix [53]. In this analysis, the dependent variable was community malaria prevalence and the independent variables were the number of battle events within 100 km since 1994 along with community averages for other variables found to be significant in the multilevel model. A regression estimate was calculated at each observation and the local parameter estimates are displayed. The results allowed us to determine if the direction of the relationship found between conflict and malaria prevalence was stationary across space.

The study was approved by the Institutional Review Board of the University of North Carolina.

## Results

### GIS mapping of malaria prevalence

**Figure 4:**
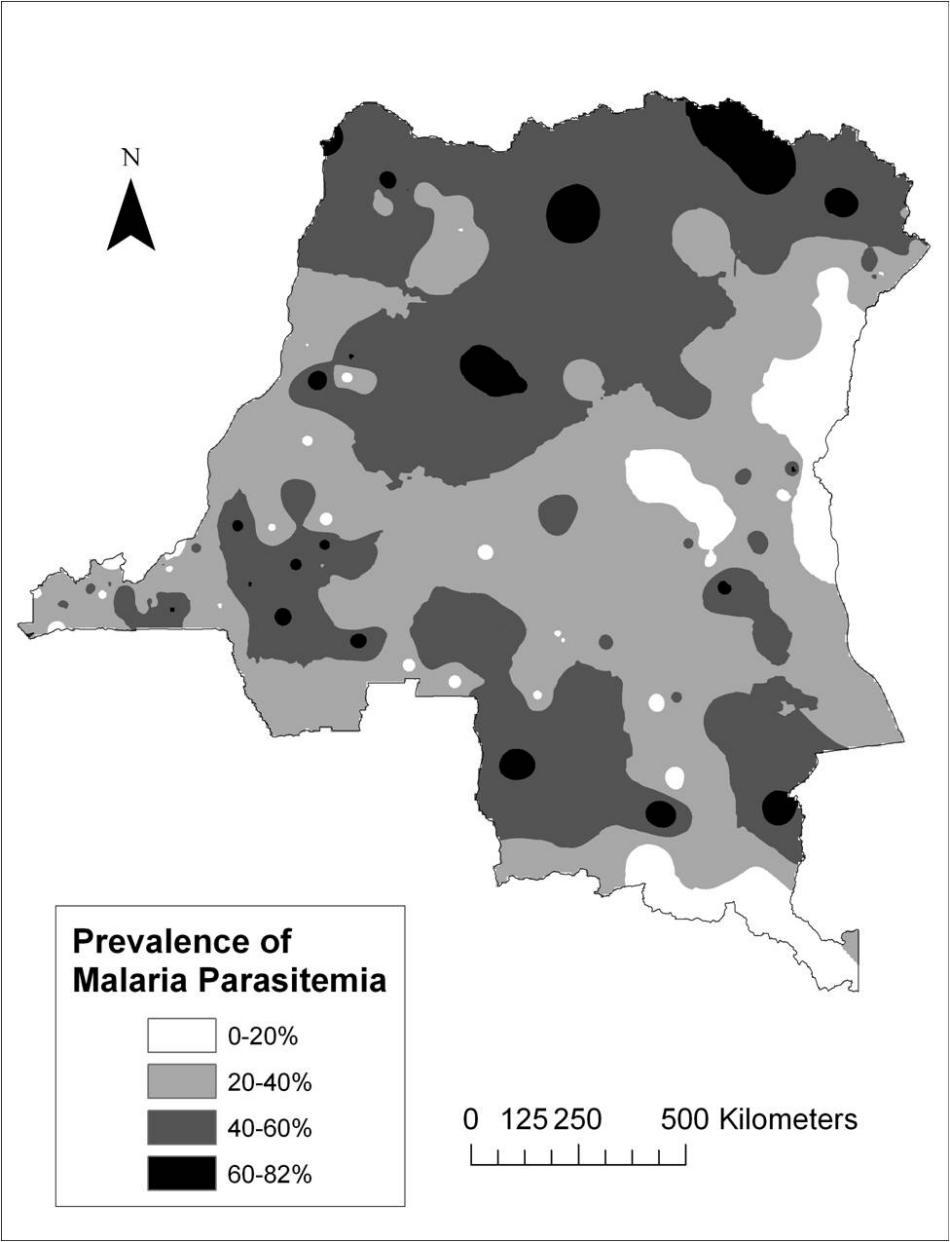
Inverse distance-weighted surface of malaria prevalence, DRC 2007.

The IDW interpolation mapping results are shown in Figure 4, with a range of 0-82% prevalence estimated across the DRC. 98% of subjects with malaria had either mono- or mixed infections with *Plasmodium falciparum *[45]. The centre and east-central regions of the country are areas of low prevalence, as well as the urban areas near Kinshasa and Lubumbashi. The northern part of the country has particularly high prevalence, as do the more rural regions near Kinshasa and Lubumbashi.

### Multivariate analysis

Table 1 shows the descriptive statistics and p-values for two-sample t-tests for unequal variances for the 29 variables entered into the initial multilevel model, as well as the hypothesized direction of association between exposure and outcome. The three conflict variables computed were highly correlated, and thus the battle events variable was chosen over rebel activity and combined conflict types since it most improved model fit. In the final model, 7746 respondents were included due to missing values for certain variables. Of these respondents, 2268 or 29.3% were parasitaemic. The final model contained 25 variables, of which 8 were statistically significant at p < 0.05. The results of this model are shown in Table 2, with statistically significant parameters highlighted in bold.

**Table 1:** Descriptive statistics for variables entered into the initial multilevel model

	Malaria-Positive	Malaria-Negative	p-value	Expected Relationship
Individual-*/Level Variables				

Age in single years - mean	28.6	30.2	<.0001	-

Male - %	52	47	<.0001	+

HIV-positive - %	1	2	0.0053	-

Education in single years -mean	5.7	6.7	<.0001	-

Wealth index (1-5) - mean	2.7	3.3	<.0001	-

Number of household members - mean	6.3	6.8	<.0001	+

Household has bed net - %	28	37	<.0001	-

Number of household bed nets - mean	.4	.6	<.0001	-

Number of kids under bed net previous night - mean	.21	.29	<.0001	-

Respondent slept under treated bed net last night - %	5.1	7.0	0.0010	-

Respondent slept under untreated bed net last night - %	10.2	15.2	<.0001	-

Child with fever past 2 weeks didn't use antimalarial - %	48	44	0.0023	+

***Community-Level Variables***				

Time to water source (minutes) - mean	31.1	26.9	<.0001	+

Time to get to health facility (minutes) - mean	72.3	53.1	<.0001	+

Distance to a town (km) -mean	45.4	30.4	<.0001	+

Distance to a city (km) - mean	139.5	106.9	<.0001	+

Distance to a river or water body (km) - mean	2.7	2.5	0.0136	+

Distance to a road (km) - mean	1.9	1.8	0.0923	+

Population density (pop/sq km) within 25 km - mean	257.9	525.1	<.0001	-

Refugee camps within 100 km of community - mean	2.5	3.3	<.0001	+ / -

Conflict events since 1994 within 100 km of community - mean	26.9	62.9	<.0001	+ / -

Within rebel territory (as defined in 2001) - %	45	45	0.7101	+ / -

Average wealth index of community (1-5) - mean	2.7	3.3	<.0001	-

Percent agricultural land cover within 10 km - mean	11.9	13.0	<.0001	+

% in community under treated net last night - mean	4.6	5.5	<.0001	-

% in community under untreated net last night - mean	9.0	12.1	<.0001	-

Altitude (km) - mean	66.8	78.2	<.0001	-

Urban community - %	34	50	<.0001	-

Accumulated rainfall, month prior to interview (mm) - mean	87.5	86.8	0.0092	+

Average air temperature, month of interview (C) - mean	24.1	23.4	<.0001	+

**Table 2:** Results of the final multilevel logistic regression model

Parameter	Beta Estimate	Odds Ratio	Lower Confidence	Upper Confidence	p-value
***Individual-Level Variables***					

**Age in single years**	**-0.0228**	**0.9775**	**0.9725**	**0.9826**	**<.0001**

Education in single years	-0.0140	0.9861	0.9711	1.0013	0.0724

**Wealth Index (1-5)**	**-0.1140**	**0.8923**	**0.8313**	**0.9577**	**0.0016**

HIV-Positive	-0.4008	0.6698	0.4040	1.1104	0.1202

**Male**	**0.2129**	**1.2373**	**1.1103**	**1.3787**	**0.0001**

Time to water source (minutes)	-0.0017	0.9983	0.9962	1.0005	0.1238

Number of household members	-0.0067	0.9933	0.9760	1.0109	0.4554

Household has bed net	0.0729	1.0756	0.8706	1.3289	0.4993

Number of household bed nets	-0.0356	0.9651	0.8663	1.0751	0.5185

Slept under treated net last night	-0.1558	0.8557	0.6602	1.1092	0.2391

Slept under untreated net last night	-0.1188	0.8880	0.7170	1.0998	0.2765

Child with fever past 2 weeks did not use anti-malarial	-0.0535	0.9479	0.8482	1.0593	0.3451

***Community-Level Variables***					

**Distance to a town (km)**	**0.0031**	**1.0032**	**1.0002**	**1.0061**	**0.0374**

Refugee camps within 100 km	-0.0068	0.9932	0.9827	1.0039	0.2114

**Number of battles since 1994 within 100 km**	**-0.0068**	**0.9932**	**0.9907**	**0.9957**	**<.0001**

**Average community wealth index (1-5)**	**-0.1714**	**0.8425**	**0.7422**	**0.9563**	**0.0080**

Percent agricultural land cover within 10 km	0.0008	1.0008	0.9997	1.0018	0.1429

Percent in community sleeping under treated net last night	-0.0054	0.9946	0.9822	1.0072	0.3988

**Percent in community sleeping under untreated net last night**	**-0.0154**	**0.9847**	**0.9758**	**0.9936**	**0.0008**

Percent agricultural land cover within 10 km	0.0007	1.0007	0.9997	1.0018	0.1592

**Altitude (m)**	**-0.0004**	**0.9996**	**0.9994**	**0.9998**	**0.0008**

Accumulated rainfall, month prior to interview (mm)	-0.0005	0.9995	0.9986	1.0003	0.2375

Avg. air temp, month of interview (C)	0.0295	1.0299	0.9814	1.0808	0.2207

Deviance					8579.83

Pearson Chi-Square					7756.28

Log Likelihood					-4289.91

AIC					8604.83

AIC for the original model was 8608.32.

Individual-level variables that significantly reduced the odds of having malaria include lower age and lower household wealth; males were nearly 24% more likely to be infected than women. Four community-level covariates reduced the odds of individual parasitaemia: altitude, the number of battles since 1994 within 100 km of a community, average wealth index of the community, and the percent of respondents in one's community having slept under an untreated bed net the previous night. While untreated bed nets were highly protective on a community level, they were not at an individual level, and insecticide-treated nets were never protective. Living further from a town slightly increased one's odds of parasitaemia.

To further examine the association between conflict and parasitaemia, a sensitivity analysis was conducted in which the communities falling within the regions of Nord-Kivu and Sud-Kivu were removed from the analysis, as these regions have experienced heavy amounts of fighting but are also characterized by higher elevations and thus less risk of malaria. Despite this, the relationship between proximity to a battle and malaria risk remained significant at p <.05.

**Figure 5:**
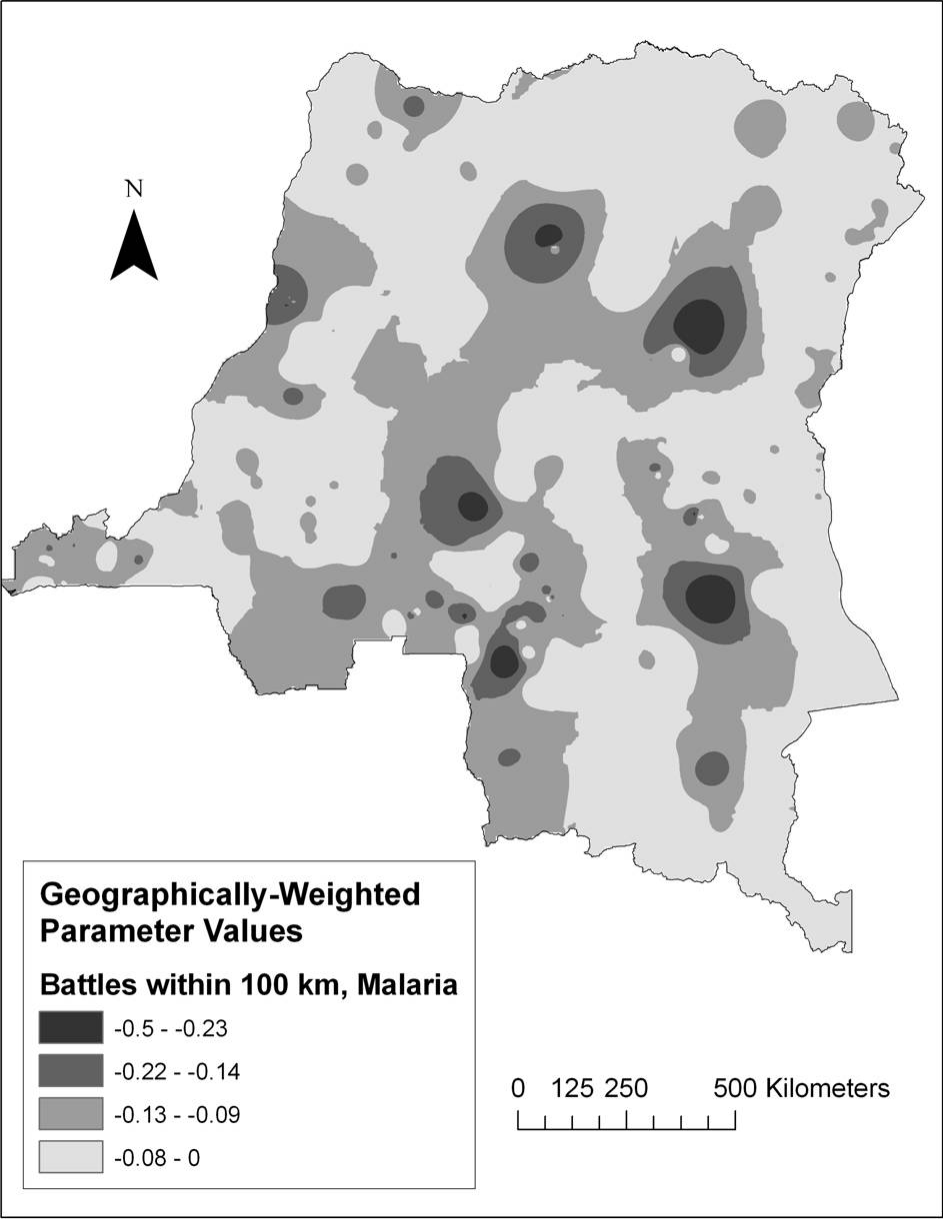
**Local parameter estimates for the relationship between 2007 malaria prevalence and the number of battles occurring within 100 km since 1994**. Areas where a positive relationship was found are highlighted in white.

The GWR analysis demonstrated the spatial distribution of the association between malaria prevalence and conflict (Figure 5). The negative association was maintained throughout the country after controlling for age, wealth, bed net ownership, gender, distance to a town, and altitude at the community level (variables found to be significant in the multilevel model).

## Discussion

The maps and model terms presented in this paper provide important insight into the factors that contribute to increased risk for malaria in certain populations and regions of the DRC. Malaria risk was found to vary geographically and to be dependent on a variety of individual-level and community-level variables. As expected, living further from a town was associated with higher rates of malaria prevalence, indicating parasitaemia is generally endemic to more rural areas. Malaria risk also decreased with increasing altitude [31-33]. Younger males were found to have the highest risk of malaria, possibly due to occupational exposures or decreased use of health care services [54]. While negative associations with malaria parasitaemia were found between rainfall and temperature, these variables were not significant after controlling for other factors.

Several factors were protective at the community level but not the individual level. Notably, living in a wealthier community more greatly decreases one's odds of having malaria than individual wealth, suggesting that less impoverished people living in impoverished neighbourhoods are still at increased risk. While individual bed net usage was significant when entered alone into the model, it was no longer significant when community bed net ownership was entered, indicating multicollinearity. Inclusion of the community bed net ownership variable provided better model fit and was thus retained. Therefore, while individual bed net ownership is important, community ownership was a stronger predictor of parasitaemia, indicating that herd immunity may be occurring within communities. Community effects on disease transmission have been reported for a variety of diseases and settings, especially for infectious diseases [55-58].

Surprisingly, the use of untreated nets was negatively associated with parasitaemia while the use of treated nets was not. While there is evidence that insecticide-treated nets are more protective against malaria [57], this finding is likely attributable to the fact that roughly twice the number of respondents slept under untreated nets as compared to treated nets (see Table 1). This is because the survey was not originally intended as a malaria indicator survey, and thus equal sampling of those with treated versus untreated nets was not a primary aim of the DHS.

Most notably, the level of conflict since 1994 occurring within 100 km of one's community was negatively associated with an individual's malaria risk in the majority of the DRC. This relationship persisted even when areas having the greatest potential to contribute to confounding (the Kivu provinces) were excluded. The GWR analysis indicated that the negative direction of the relationship was in fact stationary across the entire country.

While the relationship between conflict and infectious disease has been explored in past research [59,60], to date no studies have compared localized density of conflict with malaria parasitaemia. The inclusion of conflict variables in the models was intended to determine the outcome of parasitemia in places that have long been characterized by conflict. The nature of these places indeed differ from those areas little-affected by conflict, and this study has shown such differences to be relevant to the understanding of the drivers of parasitemia in the DRC. The inclusion of conflict density in these models is not, however, intended to indicate a direct causal relationship between specific past battle events and individual malaria parasitemia. Possible explanations for this observed association include population migration away from and increased humanitarian efforts in places having experienced large amounts of fighting. Displacement from rural areas due to conflict may lead to less dense human host populations for malaria transmission in zones of insecurity [61]. The focus of humanitarian efforts in war-affected regions on preventing and treating malaria [41] may also underlie the findings, although further knowledge of the geography and practices of humanitarian agencies in the DRC would be necessary to support this premise.

This study has several limitations. Because the presence of clinical symptoms is unknown in this study, our maps highlight where prevention may be most effective but not necessarily where treatment is most needed. Lack of blood sampling from children is also an important limitation to consider, as they suffer the greatest risk for illness and death from malaria. If children had been included in this study, malaria prevalence values might be expected to change and the significance and magnitude of parameter estimates in our models may have been different. There has only been one published report looking at age stratification of PCR-positive malaria, and the difference between adults and children was minor [62]. Data were also limited in that individuals could only be located to the centre of their community, and not to their actual place of residence. Furthermore, mobility and migration characteristics are unknown for the individuals in our dataset. Consequently, several of the geographic variables we computed (distance to a road, water body, city or town, agricultural land cover, and altitude) may lack precision and could affect the terms of these variables in the multivariate models. Finally, with the exception of Kinshasa, the study was conducted during the dry season, so the annual peak prevalence may be higher than reported here.

## Conclusions

There is much uncertainty regarding the spatial distribution of important endemic tropical diseases like malaria on the global, national, and sub-national levels. Studies have most often used passively-reported data from sentinel clinics rather than active surveillance data, leading to a bias and lack of clarity in the spatial distribution of malaria prevalence in sub-Saharan African countries. Effective resource allocation and implementation of control measures are thus hindered. While projects such as Mapping Malaria Risk in Africa (MARA) [63] and the Malaria Atlas Project (MAP) [64] have delineated endemic zones for presence of the Plasmodium parasite in sub-Saharan Africa, these projects have not employed molecular diagnostics in the DRC to determine infection in the human population. Furthermore, the Malaria Atlas Project found high levels of uncertainty in the DRC, with the fewest data points per land area of any country. Despite the limitations discussed above, this study provides the most accurate population-based estimates to date of where illness from malaria occurs in the DRC and what factors contribute to the estimated spatial patterns. In addition to increasing understanding of patterns and drivers of malaria in the DRC, this study provides an example of how population-representative surveillance can improve understanding of infectious disease prevalence and transmission.

This research demonstrates the feasibility of using population-based behavioural and molecular surveillance in conjunction with geographic methods to study endemic infectious diseases. Estimates for the prevalence of malaria are vague or unavailable in countries such as the DRC, and thus more accurate estimates of disease burden are necessary for allocating health resources. It is also important to study sub-national patterns in disease prevalence, as malaria is an infectious disease spread by the bite of an infected vector and tends to be highly geographically localized. This study suggests that spatial information and analyses can enable the DRC government to focus its control efforts against malaria.

## Competing interests

The authors declare that they have no competing interests.

## Authors' contributions

JPM performed spatial and statistical analyses as well as drafted the manuscript. SMT carried out the PCR analyses. SRM and ME participated in the design of the study. AL was involved with management of the conflict database. AKT, BA and KM helped to draft the manuscript. All authors read and approved the final manuscript.

## Funding

This work was supported by the Gillings Innovation Labs program. The NSF IGERT program at the Carolina Population Center provided support to JM.
